# Pathophysiology, Diagnosis and Treatment of Somatosensory Tinnitus: A Scoping Review

**DOI:** 10.3389/fnins.2017.00207

**Published:** 2017-04-28

**Authors:** Haúla F. Haider, Derek J. Hoare, Raquel F. P. Costa, Iskra Potgieter, Dimitris Kikidis, Alec Lapira, Christos Nikitas, Helena Caria, Nuno T. Cunha, João C. Paço

**Affiliations:** ^1^ENT Department, Hospital Cuf Infante Santo—Nova Medical SchoolLisbon, Portugal; ^2^NIHR Nottingham Biomedical Research Centre, Division of Clinical Neuroscience, School of Medicine, University of NottinghamNottingham, UK; ^3^Centro em Rede de Investigação em Antropologia (CRIA), Network Centre for Research in Anthropology, Universidade Nova de LisboaLisbon, Portugal; ^4^First Department of Otorhinolaryngology, Head and Neck Surgery, National and Kapodistrian University of Athens, Hippocrateion General HospitalAthens, Greece; ^5^Institute of Health Care, Mater Dei HospitalMsida, Malta; ^6^Deafness Research Group, BTR Unit, BioISI, Faculty of Sciences, University of LisbonLisbon, Portugal; ^7^ESS/IPS–Biomedical Sciences Department, School of Health, Polytechnic Institute of SetubalLisbon, Portugal; ^8^ENT Department, Hospital Pedro Hispano—MatosinhosLisbon, Portugal

**Keywords:** somatosensation, somatosensory, tinnitus, physical therapy, physiotherapy, cross modal

## Abstract

Somatosensory tinnitus is a generally agreed subtype of tinnitus that is associated with activation of the somatosensory, somatomotor, and visual-motor systems. A key characteristic of somatosensory tinnitus is that is modulated by physical contact or movement. Although it seems common, its pathophysiology, assessment and treatment are not well defined. We present a scoping review on the pathophysiology, diagnosis, and treatment of somatosensory tinnitus, and identify priority directions for further research.

**Methods:** Literature searches were conducted in Google Scholar, PubMed, and EMBASE databases. Additional broad hand searches were conducted with the additional terms etiology, diagnose, treatment.

**Results:** Most evidence on the pathophysiology of somatosensory tinnitus suggests that somatic modulations are the result of altered or cross-modal synaptic activity within the dorsal cochlear nucleus or between the auditory nervous system and other sensory subsystems of central nervous system (e.g., visual or tactile). Presentations of somatosensory tinnitus are varied and evidence for the various approaches to treatment promising but limited.

**Discussion and Conclusions:** Despite the apparent prevalence of somatosensory tinnitus its underlying neural processes are still not well understood. Necessary involvement of multidisciplinary teams in its diagnosis and treatment has led to a large heterogeneity of approaches whereby tinnitus improvement is often only a secondary effect. Hence there are no evidence-based clinical guidelines, and patient care is empirical rather than research-evidence-based. Somatic testing should receive further attention considering the breath of evidence on the ability of patients to modulate their tinnitus through manouvers. Specific questions for further research and review are indicated.

## Introduction

Tinnitus is defined as the conscious perception and reaction to a sound in the absence of a matching external acoustic stimulus, commonly described as a *phantom* perception. It is considered a symptom rather than a disease *per se* (Jastreboff and Hazell, 1993; Bürgers et al., 2013). Tinnitus is present in more than 10% (11.9–30.3%) of the adult population (McCormack et al., 2016), although only 0.5–3% refers to it as a problem that decreases quality of life (Coles, 1984; Swain et al., 2016).

Although tinnitus has been the subject of much research, its pathophysiology remains poorly understood. It is well-accepted that many social factors, such as poor education, lower income, or occupational and recreational activity associated with high noise exposure, influences the prevalence and risk of tinnitus (Hoffman and Reed, 2004). Moreover, it is regularly associated with hearing loss, trauma, or ototoxic medication triggering cochlear damage, with sustained neural changes in the central auditory system that succeeds such lesions (Møller, 2011a; Langguth et al., 2013). Tinnitus prevalence is believed to increase with age up to 65 years, where after it decreases (Hoffman and Reed, 2004; Shargorodsky et al., 2010). It is also a widespread symptom among children with hearing loss (Coelho et al., 2007) and many causes of hearing loss and tinnitus are thought to be the same (Crummer and Hassan, 2004).

Recent neuroimaging and animal model studies suggest that tinnitus-related neural activity may involve complex interactions between several sensory modalities, sensorimotor, somatomotor, and visual-motor systems, neuro-cognitive, and neuronal-emotional networks (Cacace, 2003; Sanchez and Rocha, 2011a,c; Ostermann et al., 2016). Signs of interactions between the auditory system and the somatosensory system include gaze-evoked tinnitus (Cacace et al., 1994; Pinchoff et al., 1998; Lockwood et al., 2001), cutaneous-evoked tinnitus (Cacace et al., 1999a,b), motor manipulation or forceful muscle contractions of head, neck and limbs that induce or suppress tinnitus, or affect tinnitus loudness (Sanchez et al., 2002, 2007; Simmons et al., 2008). Pressure on myofascial trigger points (Travell, 1960; Wyant, 1979; Fricton et al., 1985; Bjorne, 1993; Rocha et al., 2006, 2008; Rocha and Sanchez, 2007), electrical stimulation of the median nerve and hand (Moller and Rollins, 2002), finger movements (Cullington, 2001), orofacial movements (Pinchoff et al., 1998), and pressure applied to the temporomandibular joint (i.e., Bjorne, 1993) are also observed to modulate tinnitus in some people. Such “somatosensory tinnitus” is supposed to be a prevalent tinnitus subtype (for review see Ralli et al., 2016) and prevalence may even be under-estimated because it relies on self-report that tinnitus is modulated by touch or movement (Ward et al., 2015). For example, the prevalence of somatic modulation is higher when the patients are questioned specifically about it rather than spontaneous reports (Sanchez et al., 2002).

For clarity we will use the following definitions: Tinnitus Modulation is the human capability of changing the tinnitus perception (frequency or intensity) by means of performing a certain manouver or movement of the head or neck or jaw or limbs or the eyes. Triggers is the phenomenon that acivates tinnitus modulation, examples: gaze movement, some tactile stimulous, performing a certain manouver or movement of the head or neck or jaw or limbs or the eyes. So the peripheral activity or stimulation are the primary single sources of a precise modulation of the tinnitus sound and it is described as trigger activity and the term modulation is reserved solely for describing the central neural activity that affect changes in tinnitus percept.

In the most comprehensive literature review to date on somatosensory tinnitus, Sanchez and Rocha (2011a,b) spoke of the need to establish evaluation protocols and specific treatments for somatosensory tinnitus that focus on both the auditory pathway and the musculoskeletal system. Yet there has never been a scoping review or systematic review on the topic. In this review, we scope the primary research literature on the pathophysiology, diagnosis, and treatment of somatosensory tinnitus. The aims of the review are to account the breadth and current state of knowledge on somatosensory tinnitus, to consider priority directions for research, and to identify whether any systematic reviews would be informative to the field.

## Materials and methods

Literature searches were conducted in November 2016 in Google Scholar, PubMed, and EMBASE databases using the search terms somato^*^ AND tinnitus (see Appendix 1 in Supplementary Material for an example search). Search results were screened to identify original articles and case reports for review. For Google Scholar, results were screened until five consecutive results pages yielded no new potentially relevant results. Additional hand searches of publications were conducted in the same databases using the additional broad search terms etiology, diagnose, treatment. Records were independently reviewed by at least two authors. In cases of disagreement, opinion of a third reviewer was taken as consensus. Inclusion criteria were: somatosensory tinnitus as main or secondary study objective, inclusion of at least one group with patients or case study suffering from somatosensory tinnitus, definition of somatosensory tinnitus, description of somatosensory tinnitus diagnostic approach or treatment. If the focus of the study was somatosensory tinnitus pathophysiology, diagnosis, or management, and at least one of the study groups or case study consisted of somatosensory tinnitus patients, the study was included; otherwise it was excluded. Exclusion criteria were articles written in languages other than English, and records relating solely to objective tinnitus.

**Figure 1:**
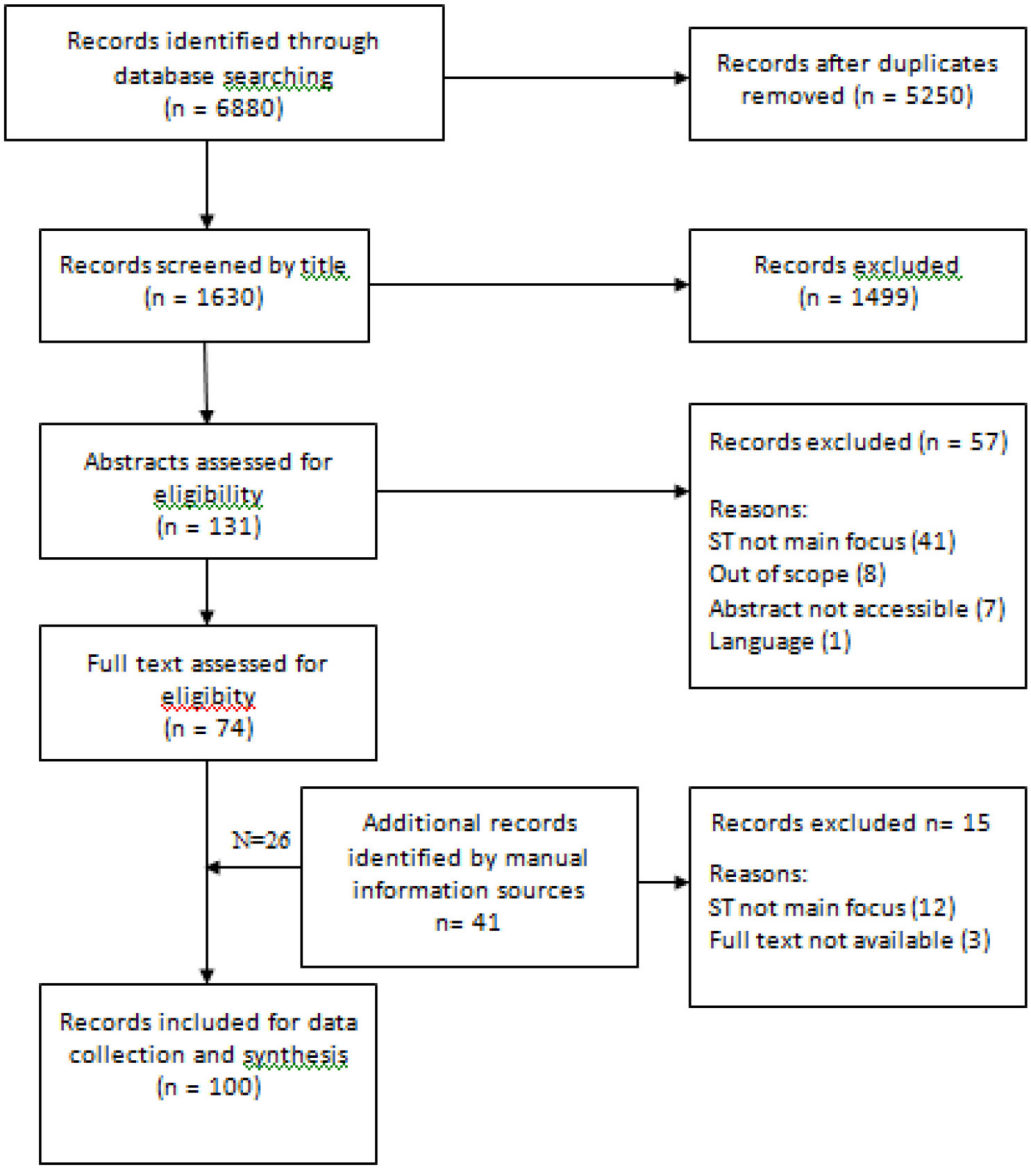
**Flow chart of study records**.

Initial screening was based on abstract reading. Where there was uncertainty whether or not a record was relevant the full text record was screened. Records were grouped into three categories: pathophysiology, diagnosis, and treatment. One record could be relevant to more than one category. All records included patients with somatosensory tinnitus (P). Interventions (I) and their effects were recorded. Outcome measures were also identified (O), and comparisons (C) were described either between patients and controls, groups of patients divided by tinnitus type or intervention, as well between groups of patients before and after intervention for somatosensory tinnitus (see Figure 1).

## Results

The initial searches for somato^*^ AND tinnitus yielded 1,630 records of which 100 were suitable for inclusion in the review. Records are subdivided for review according to pathophysiology, diagnosis, and management.

### Pathophysiology and etiology

Records describing studies on the pathophysiology and etiology of somatosensory tinnitus were included and are reviewed here. A table compiling the case controlled studies and cross-sectional studies were summarized in Appendix 2 in Supplementary Material (case reports, reviews and book chapters were excluded).

A number of authors suggest the somatosensory stimuli inducing tinnitus are deeply related to abnormal cross-modal plasticity of somatic-auditory interactions (Cacace, 2003; Levine et al., 2007; Herraiz, 2008; Rocha et al., 2008; Koehler and Shore, 2013) whereby somatic modulations of tinnitus results from abnormal auditory neural interactions—distortion of the normal synaptic activity—within the central nervous system, as Sanchez et al. (2007) describes, “*The information triggered by muscle contractions is carried by the somatosensory system and, upon reaching the cuneiform nucleus, may influence tinnitus through its projection over the auditory pathway due to an overactivitiy in the cochlear nucleus.”* In particular, modulation of hyperactivity of neurons in the dorsal cochlear is triggered by the stimulation of specific ipsilateral cranial nerves, i.e., branch of the trigeminal nerve, explaining how ipsilateral tinnitus may be modulated by head and neck's manipulation (see a review, Kaltenbach, 2006). In guinea pigs, it was demonstrated that DCN bimodal plasticity is stimulus timing-dependant and implicated as an underlying mechanism in tinnitus (Shore et al., 2007; Koehler and Shore, 2013).

Levine et al. (2003) found somatic modulation in patients with tinnitus and deafness patients, identifying neural interactions in the central nervous system as the main protagonists in this process. Levine et al. (2008) also suggest that pulsatile tinnitus is modulated by the somatosensory system of the head or upper lateral neck, presenting two mechanisms; (1) cardiac synchronous somatosensory activation of the central auditory pathway, or (2) distortion of the normal synaptic activity between the somatosensory and auditory central nervous system. Simmons et al. (2008), studying patients who could modulate tinnitus with jaw clench found that an alteration in tinnitus loudness related to a variation in neural activity in the auditory cortex, concluding that tinnitus originates in the central auditory pathway. The same effect has been observed in patients who can modulate their tinnitus with eye movements (Lockwood et al., 2001; Sanchez and Akemi, 2008), and in patients whose tinnitus is modulated by intravenous administration of lidocaine (Reyes et al., 2002). Modulation of tinnitus with oral-facial movements suggest that the classical auditory system is not implicated in tinnitus because limbic structures respond to sound stimulation in patients with tinnitus (through hypoactivity localized in the hippocampus), further indicating the central auditory system and not the cochlea as the origin of tinnitus (Lockwood et al., 1998; Cacace, 2003; Schaette and McAlpine, 2011). In his studies, Levine found that patients could better detect changes in their tinnitus when using isometric maneuvers of the extremities, compared to head/neck maneuvers, suggestive of a major role of the central neural pathway as opposed to the auditory periphery (Cacace, 2003). In fact, a higher prevalence of somatoform disorders in individuals with tinnitus may also relate to certain craniocervical pathological features (e.g., herniated discs or temporomandibular joint syndrome; Chole and Parker, 1992; Rubinstein, 1993; Gelb et al., 1997; Levine, 1999b) and dental and jaw diseases (Han et al., 2009). For example, there is a higher than general incidence of tinnitus in patients and normal hearing who have temporomandibular disorder (TMD) (Levine, 1999b), suggesting that it may be associated with other symptoms of TMD (Chole and Parker, 1992; Bernhardt et al., 2011). The temporomandibular joint (TMJ) is thought to be commonly involved in the ability to modulate tinnitus, particularly its loudness (Ralli et al., 2016). Recently the risk of tinnitus was established as 8.37 times higher for patients with TMD (Bürgers et al., 2013), and unilateral tinnitus is even reported to be on the same side as unilateral TMD (Bürgers et al., 2013). These patients are also reportedly able to regulate their tinnitus through certain jaw or neck movements (Wright and Bifano, 1997a; Vielsmeier et al., 2011, 2012; Bürgers et al., 2013). Since tinnitus is normally related to the opposite risk factors (i.e., older males with hearing loss), such findings postulate that TMJ may be the cause and maintenance of tinnitus (Vielsmeier et al., 2011). It is proposed that TMD can cause tinnitus through the disruption of the trigeminal input (Vielsmeier et al., 2012; Ostermann et al., 2016). Another indication supporting the role of TMD in tinnitus is that the two conditions occur simultaneously. Evidence also shows that worsening of tinnitus coincides with aggravation of TMD (Wright and Bifano, 1997b).

### Diagnosis

Records describing studies on diagnosis or rate of diagnosis of somatosensory tinnitus were included and are reviewed here. A table compiling the case controlled studies and cross-sectional studies were summarized in Appendixes 3, 4 in Supplementary Material (reviews, thesis, and book chapters were excluded), concerning both epidemiology and diagnosis fields, respectively.

Common attributed risk factors for any subtype of tinnitus are male gender, older in age and hearing problems (i.e., Hazell, 1991; Abel and Levine, 2004; Eggermont and Roberts, 2004; Hoffman and Reed, 2004; Oostendorp et al., 2016), except for TMD-tinnitus patients (Chole and Parker, 1992; Wright and Bifano, 1997b; Vielsmeier et al., 2011; Bürgers et al., 2013). Recent evidence in a British cohort study shows that somatic tinnitus is more common among younger people and it is unrelated to hearing loss or tinnitus severity (Ward et al., 2015). Some of these audiological and demographic traits, may be indeed useful in informing therapy (Won et al., 2013) through the identification of “clinical criteria for useful subtyping of tinnitus patients” (Vielsmeier et al., 2012).

Signs of somatosensory tinnitus include head or neck problems (i.e., temporomandibular joint syndrome, osteophits, arthorosis, spondylosis, myofascial trigger points, etc.), dental or jaw diseases, frequent pain in head, neck, or shoulder girdle, aggravation of events of simultaneous pain and tinnitus, incorrect body postures, and severe bruxism (Sanchez and Rocha, 2011b,c). Such complexity demands a multidisciplinary team (i.e., dentist, physiotherapist) to diagnose.

Somatosensory tinnitus is strongly evidenced when the patient can modulate the loudness or intensity of their tinnitus (Abel and Levine, 2004; Latifpour et al., 2009; Sanchez and Rocha, 2011b,c; Oostendorp et al., 2016). Hence somatic testing may identify patients who could be treated with somatosensory system-related therapies. However, this type of testing receives little attention (Won et al., 2013).

There are various presentations of somatosensory tinnitus to be aware of. Typical cases include gaze-evoked or modulated tinnitus, cutaneous-evoked tinnitus, and tinnitus modulated by movement of corporal elements (i.e., head, fingers, jaw). Gaze-evoked/modulated tinnitus, the modulation of tinnitus by eye movement, provides clues on the potential cortical role in tinnitus (Lockwood et al., 2001). Simmons et al. (2008) found a large sample of patients who were capable of modulating their tinnitus by eye movement, half of whom had developed this ability after undergoing surgery for removal of an acoustic neuroma; these patients were able to change the tinnitus loudness and pitch through eye movement.

Studies of cutaneous-evoked tinnitus (using magnetoencephalographic signals and tactile discrimination tests) have found that cutaneous stimulation of skin on the hand region (specifically palm and fingers) activates the somatosensory system along with the auditory cortical areas in congenitally deaf individuals (Cacace et al., 1999a,b; Cacace, 2003).

In respect to modulation of tinnitus through of head and neck, Levine (1999a) reported that 68% of 70 patients could modulate tinnitus through maneuvers of the head, neck, or less intensely, maneuvers of limb. Similarly, Sanchez et al. (2002) found both patients with tinnitus (65.3% of 121 persons) and healthy subjects (14% of 100) could modulate or develop, respectively, tinnitus through 16 different maneuvers, and later found 57.9% of a study population could modulate tinnitus using nine different maneuvers (Sanchez et al., 2007). Simmons et al. (2008) found that, in 93 subjects able to modulate tinnitus by jaw clench, 90% could increase the loudness of their tinnitus, and 50% could alter the pitch. In a different assessment, the same authors found that 78% of their sample of 45 subjects could modulate their tinnitus with movement of the head or neck, mainly using the cranial and cervical nerves and using forceful maneuvers. In another study, Won et al. (2013) found that in 57% of tested ears in a population sample of 163 patients, tinnitus (especially unilateral tinnitus) was modulated through neck maneuvers or jaw maneuvers, decreasing and increasing tinnitus loudness respectively. The authors also reported that in their sample bilateral and low-pitch tonal tinnitus was rarely modulated by movement and may even be aggravated by somatic therapy. More distal movement is also observed to modulate tinnitus. Cullington (2001) reported the case of a 78-year-old man with severe hearing loss implanted with a cochlear implant in his right ear was able to modulate his tinnitus by moving his finger. Fascinatingly, this patient reported that the quicker the movement, the more intense was tinnitus loudness; passive or isometric movement did not modulate the tinnitus (Sanchez and Akemi, 2008). See Table 1 for a summary of somatic maneuvers.

**Table 1:** **Summary of somatic manouvers**.

**Authors**	**Body part**	**Maneuvres (examples)**
Cullington, 2001	Finger	Moving up and down the middle finger of left hand^**^^#^
Levine, 1999a; Sanchez et al., 2002, 2007; Abel and Levine, 2004; Levine et al., 2007	Extremities	Locking the fingers of the two hands together and pulling as hard as possible, or resisting maximal pressure to. Shoulder abduction. Flexion or abduction of the hip. Resisting or not an applied force.
Lockwood et al., 2001; Sanchez and Akemi, 2008; Simmons et al., 2008	Eye	Moving in the vertical or horizontal axis^**^
Cacace et al., 1999a,b; Cacace, 2003; Sanchez and Akemi, 2008	Cutaneous	Stimulation of a well-defined region—various regions of the hand and fingers (e. g., palm, dorsal web regions, and fingertips)^**^^&^
Pinchoff et al., 1998; Sanchez et al., 2002, 2007; Abel and Levine, 2004; Levine et al., 2007; Simmons et al., 2008; Latifpour et al., 2009; Won et al., 2013	Jaw	Clench the teeth, open and close mouth, protrude jaw, slide jaw. Resisting or not an applied force.
Levine, 1999a; Sanchez et al., 2002, 2007; Abel and Levine, 2004; Simmons et al., 2008; Latifpour et al., 2009; Won et al., 2013	Head and neck	Moving the head back and in front and laterally, resisting or not an applied force (against the head in a neutral position or turned to one of the sides).
		Applying pressure on muscle insertions–esternocleidomastoid, splenius capitis, and posterior auricular.

*All the different voluntary muscle contraction manouvers should be sustained during 5–10 s and performed using a moderate degree of force in a silent environment (Levine, 1999a)*.

*The idiopathic somotosensorial tinnitus will present more relevant modulation with jaw and head-neck manouvers*.

** *Very specific to certain cases of patients subjected to brain neurosurgery or cochlear implantation only rarely is it spontaneous*.

# *The patient reported that the quicker the movement, the more intense the tinnitus loudness, passive or isometric movement did not modulate the tinnitus*.

& *Studies of cutaneous-evoked tinnitus, (using magnetoencephalographic signals and tactile discrimination tests) have found that electrical stimulation of the median nerve and hand region or cutaneous stimulation of skin on various regions of the hand including dorsal web regions and fingertips activate the somatosensory system along with the auditory cortical areas in congenitally deaf individuals (Cacace et al., 1999a,b; Cacace, 2003)*.

Even when the patient cannot self-modulate tinnitus, it may be altered by other kinds of stimuli, using maneuvers to increase activity of the trigeminal nerve such as passive muscular palpation to find myofascial trigger points (MFT), relaxation, and massage (Simmons et al., 2008; Sanchez and Rocha, 2011b; Shore, 2011; Won et al., 2013).

### Treatment

Records describing studies on the treatment of somatosensory tinnitus were included and are reviewed here by treatment category. Case controlled studies and cross-sectional studies were summarized in Appendix 5 in Supplementary Material.

### Physiotherapeutic treatment

Studies have accounted the benefits for tinnitus of treating (temporomandibular disorder) TMD. Wright and Bifano (1997a) studied tinnitus in TMD patients and reported that 56% had been cured and 30% had a significant improvement with cognitive therapy and modulation through maneuvers. However, it has also been found that that severe tinnitus is less likely to improve with TMD therapy (Wright and Bifano, 1997a). Another similar study has shown that younger patients with moderate tinnitus were more likely to experience relief of their tinnitus through TMD therapy (Wright and Bifano, 1997b). Tinnitus severity as a predictor of the effectiveness of TMD therapy has already been proposed by others including Erlandsson et al. (1991) and Bush (1987).

The presence of fluctuating tinnitus is another factor that may associate with TMD treatment effectiveness (e.g., Tullberg and Ernberg, 2006).

One form of TMD treatment is occlusal splint therapy (Attanasio et al., 2015). In their study involving this treatment in patients presenting with chronic subjective tinnitus Attanasio et al. (2015) divided patients into three groups according to whether TMD was absent, present, or the patient was considered predisposed to TMD. Patients were subjected to treatment with a neuromuscular occlusal splint for 6 months (using the splint at night time) and rated for the severity of tinnitus using 10-point visual analog scale and Tinnitus Handicap Inventory (THI; Newman et al., 2004) questionnaire. Post-treatment THI scores were reduced in all groups but was most pronounced in the TMD (experience or predisposed) groups. The authors concluded that, once otologic disorders and neurological diseases are excluded, that clinicians should refer patients for an evaluation of the temporomandibular joint and subsequently to treat patients with TMD or a predisposition to it.

Wright (2000) suggested oro-myofunctional therapy as an effective alternative to occlusal splints therapy. Their study involved patients from the US air force seeking treatment for tinnitus, dizziness, and/or nonotologic otalgia without an identifiable cause and presenting with TMD symptoms in the temple, jaw, or preauricular area. Patients were provided a dental orthotic and TMD self-care instructions. After 3 months of orthotic wear, the percentages of patients reporting at least moderate symptom improvement of their tinnitus, dizziness, otalgia, and/or TMD were 64, 91, 87, and 92%, respectively. Follow-up telephone calls 6 months after completion of TMD therapy revealed that all patients maintained their symptom improvements. These findings imply that TMD was affecting the patients' otologic symptoms.

### Stomatognathic therapy

Usually it includes splints therapy, therapeutic exercises for the lower jaw and occlusal adjustment in combination with counseling.

For a long time, scientists have investigated the effects of dental and stomatognathic therapies in tinnitus (Junemann, 1941; Gelb and Arnold, 1959; Dolowitz et al., 1964; Kelly and Goodfriend, 1964; Gelb et al., 1967; Koskinen et al., 1980; Ioannides and Hoogland, 1983; Cooper et al., 1986; Bush, 1987; Rubinstein and Erlandsson, 1991). According to the findings of Rubinstein (1993), almost one-third of patients report improvement in their tinnitus after mandibula movements and/or pressure on their TMJs. More recently, Bürgers et al. (2013) found that stomatognathic therapy had a positive effect on tinnitus symptoms in 44% of their TMD-tinnitus patients (*n* = 25), up to 3–5 months after the first intervention; while promising it is noted that there was no control group in this study. Using dental functional therapy, the authors found an improvement on acute or subacute tinnitus in 100% of the patients but little improvement in patients with chronic tinnitus. It is important to note that the authors discussed an individual therapeutic strategy with each patient before the start of treatment. The authors suggested long term studies are conducted to assess the outcome and advised caution when interpreting current epidemiological data.

### Chiropractic therapy

Chiropractic therapy is a correction therapeutic treatment of an abnormal movement pattern through the manipulation of the vertebral column and extremities. Only three studies related to chiropractic treatment of tinnitus were identified and all three were case studies. Alcantara et al. (2002) described the chiropractic therapy in a 41-year-old woman with history of ear pain, tinnitus, vertigo, altered hearing, ear infections, and headaches, and who was diagnosed TMD and cervical subluxation. The authors reported a complete relief from the TMD symptoms, including tinnitus, after only 9 treatments (2 months). The treatment involved the application of high-velocity low amplitude adjustments. Kessinger and Boneva (2000) also reported progress in a 75-year-old patient who received upper cervical specific chiropractic care which resulted in improvements in vertigo, tinnitus, and hearing loss. These authors concluded that the success of chiropractic therapy was due to improvement in cervical spine function.

DeVocht et al. (2003) also describes the chiropractic management of a 30-year-old woman with TMJ pain. The patient suffered daily from unremitting jaw pain for 7 years accompanied by headache, tinnitus, decreased hearing, and a feeling of congestion in her right ear. Twenty months of chiropractic treatment resulted in total resolution of all symptoms except fullness of the right cheek.

### Muscle relaxation

Combined with chiropractic care, muscular relaxation (through massage and stretching exercises) is used in clinical practice. For instance, evidence suggests that palpation of masseter, pterygoid, and sternocleidomastoid muscles or myofascial trigger points can modulate tinnitus (Rocha et al., 2008; Teachey et al., 2012). Björne (2007) reported on the effectiveness of stretching exercises targeting the suboccipital muscles, along with rotation movements in the atlanto-occipital joint and relaxing exercises, on a TMD patient population (no control group). Bjorne notes that patients with Ménière's were more likely to present with TMJ and cervical spine disorder's symptoms (including tinnitus), than people who do not have Ménière and using a coordinated therapy of TMJ and cervical spine disorder (relaxation and posture) found improvements in self-reported tinnitus severity that were retained up to 3 year follow up.

Latifpour et al. (2009) evaluated 24 subjects from an original pool of 41 subjects (non-randomized), divided into two groups: treatment and control group. The authors compared self-training of stretching, posture training, and acupuncture, targeting muscle symmetry and balance in the jaw and neck, and later reported an improvement of tinnitus in the treatment group. In this blinded study they observed immediate and long term (3 months) improvements in the treatment group.

Another therapy worth noting here; in a pilot study with 11 patients, Kaute (1998) reported improvement in vestibular disturbances through the method of Arlen's Atlas Therapy, normally applied to whiplash-injured patients, concluding it to be indicated where tinnitus may be caused by neck muscle tension. This study suggest that muscular relaxation may play a significant role in the treatment of tinnitus but high quality explanatory studies (i.e., comparison with a control, blinded, randomized allocation), are needed.

### Somatic modulation therapy

Somatic modulation therapy (treatment aiming to modulate the intensity of a given symptom, by movement) has rarely been studied beyond case studies. Sanchez et al. (2007) were the first to investigate the effect of repetitive training maneuvers with head and neck muscle contractions, focusing on its value as a tinnitus retraining therapy. The authors found it to have a significant effect on the modulation patterns but not in the daily perception of tinnitus.

In the case of a 39-year-old woman who developed gaze-evoked tinnitus after surgery to remove a left vestibular Schwannoma, therapy consisted of a repetitive gaze training and tinnitus was resolved after 14 weeks (Sanchez and Akemi, 2008). Interestingly, there was both a “horizontal” and “vertical” gaze effect on tinnitus and the vertical component responded more quickly to treatment suggesting more than one neural network or process was involved in this case.

In another case, a 54-year-old man with severe tinnitus noticed an improvement through tactile stimuli to the ipsilateral postauricular area, head rotation, opening of the mouth, and clenching teeth and mandible lateralization (Sanchez and Akemi, 2008). In another case of tinnitus improvement through tactile stimulation was reported in a single patient by Emmert et al. (2014); the patient reported a decrease in tinnitus intensity in the left ear when a tactile stimulus was applied (block-design using EPI sequence—the patients touched on the right cheek on seven blocks of 25 s, intercalating with 25 vs rest).

### Electrical stimulation

Recent evidence reported a significant improvement in tinnitus using transcutaneous electrical nerve stimulation (Herraiz et al., 2007; Vanneste et al., 2010). Trans-electrical nerve stimulation (TENS) of areas of skin close to the ear increases the activation of the dorsal cochlear nucleus through the somatosensory pathway and may augment the inhibitory role of this nucleus on the CNS and thereby ameliorate tinnitus (Herraiz et al., 2007).

Vanneste et al. (2010) applied transcutaneous nerve stimulation in the upper cervical nerve in 240 patients with the ability to modulate tinnitus and found a significant suppression of tinnitus. Although only 18% of the patients responded to the treatment, 43% declared an improvement and six patients reported a total suppression of tinnitus (Vanneste et al., 2010). Herraiz et al. (2007) showed that trans-electrical nerve stimulation led to improvements in 46% of somatic tinnitus patients (reduced VAS tinnitus severity scores) after 2 weeks of treatment. Intermittent “typewriter”—sound like tinnitus was the most responsiveness. Herraiz et al. (2007) also noted that tinnitus caused by a somatosensory injury had a better response than somatic tinnitus with an otologic disease.

Standardizing the indications and method could increase the efficacy of electrical stimulation in somatic tinnitus according to most authors. These results are promising so further controlled trials are warranted.

### Pharmaceutical treatment

Only one relevant record describing a pharmaceutical treatment was included. In this case study McCormick and Walega (2015) reported the successful treatment of refractory somatic tinnitus with cervical epidural injection of 80 mg triamcinolone acetonide. The patient was 61-year-old male with previous history of bacterial otitis media.

### Surgical treatment

No surgical treatment studies specific to somatosensory tinnitus were identified. One case study worth mentioning however was that of a 65 years old patient with left sided tinnitus and with left sided cervical neck pain who experienced a complete resolution of somatic tinnitus for over 1 year through radiofrequency ablation of the left C2–C3 medial branches of the dorsal ramus ipsilateral to tinnitus symptoms (Gritsenko et al., 2014).

## Discussion

Tinnitus is complex in nature and so ideally, and to achieve the best results, diagnosis and treatment should be specific to an individual patients experience. Further research on the physiological processes that lead to somatosensory tinnitus would facilitate the development of a specific protocol and therapy targeting the auditory pathways and musculoskeletal disorders (Sanchez and Rocha, 2011c). Indeed, any holistic view of tinnitus needs to take into consideration the auditory system as a dynamic and active structure, integrating systems of reaction, stimulation, and emotion and tinnitus itself as a symptom with complex causes that indicate hyperactive neural activity (Møller, 2011a) and activation of neural plasticity (Moller and Rollins, 2002; Møller, 2011b; Smith et al., 2013), without the participation of the ear (Møller, 2016).

Evidence points to a high prevalence of somatosensory tinnitus, but that it is under-investigated by clinicians and the processes underlying are still poorly studied. For instance, only very recently have the first steps been made toward understanding the genetic underpinnings of subjective tinnitus (Lopez-Escamez et al., 2016) or the social context and environment which may influence tinnitus, following the new Social-Neurophysiological Model of Tinnitus. This model proposes the integration of the neurophysiological system (Jastreboff, 1990; Jastreboff and Jastreboff, 2000) the relation between psychophysiological and behavioral systems) and the social information system, associated with the emotional experience of tinnitus (Li et al., 2015). These avenues may help develop clinical strategies that adapt to patient's understanding and attitudes toward tinnitus, through social learning. What these will mean for somatosensory tinnitus is an open question.

It is important to note that an early and precise diagnosis, presents the best outcomes for the patient treatment (Herraiz, 2008). Recent research on the treatment of somatosensory tinnitus has focused on bone and muscular disorders, on each structure independently or using multimodal approach including manual therapy and exercise (Michiels et al., 2014, 2016). This demands different practitioners (dentists, neurologists, audiologists, physiatrist etc.) to be involved in treatment. Although such strategies do not target tinnitus directly, such therapies are shown to ameliorate its side effects.

It is not possible to cure tinnitus through dental and TMD therapies. But these same therapies may contribute to a multidisciplinary methodology of tinnitus treatment (Herraiz, 2008; Bürgers et al., 2011). It is a priority to establish how TMD and somatosensory tinnitus are related and what criteria should be used to select tinnitus patients for different TMD therapies. Further research is needed to attest the efficacy of TMD therapy on tinnitus and to access the placebo effect (Rubinstein, 1993; Tullberg and Ernberg, 2006).

A multidisciplinary approach to managing somatosensory tinnitus may result in different strategies being used by different teams of clinicians if there is poor interdisciplinary communication and the lack of large-scale controlled trials to inform evidence-based clinical guidelines (Møller, 2007). In addition, standardization of core measures hinders the process of any potential meta-analysis on the large datasets, which would aid the development of clinical interventions for tinnitus. However, it will need to be tested whether these standardized outcomes are sensitive to treatment related changes in groups of patients or trail participants who have somatosensory tinnitus.

## Conclusion

Because somatosensory tinnitus is not judged a disease *per se*, but instead it is considered a symptom, its diagnosis and treatment were related to other disorders. Connection to hearing loss and bone and muscular disorders are evident.

With this scoping review, we intended to give the reader a broad overview of findings to date concerning somatosensory tinnitus, and encourage new systematic and integrative analyses which will hopefully bring the much-needed order to the field of tinnitus research.

We propose several outstanding studies on somatosensory tinnitus:

1. There is some discrepancy over the prevalence of somatosensory tinnitus; a systematic review is needed.
2. The etiology of somatosensory tinnitus needs continued investigation. Particularly, and considering the involvement of neural plasticity, it is necessary to determine the exact processes that initiate the abnormal cross-modal plasticity of somatic-auditory interactions. Moreover, it is important to determine the exact relation between the head/neck maneuvers in the central neural system.
3. There is a lack of objective diagnostic methodology, which may misguide clinical management. Clinical guidelines that consider or are specific to somatosensory tinnitus are needed.
4. There are many and different strategies for managing tinnitus, originating in different clinical fields (audiology, neurology, psychology, etc.), and not all strategies have been trialed in somatosensory tinnitus. Integrating such strategies, and having in mind that each patient is a singular case, may increase the success of clinical management practices for tinnitus.
5. To support further trials and data synthesis in somatosensory tinnitus there needs to be standard research methodologies. Theses should be developed through consensus.
6. A therapeutic intervention combining simultaneously several types of treatment approaches may bring the best results for tinnitus relief, but such combinations may also be individual specific.

## Author contributions

HH is the guarantor of the review. DH and DK created the search strategies. DK and CN created the tables in appendix. IP contributed in data extraction and initial manuscript. HH, DH, and RC contributed equally to all other stages of the manuscript development, produced, and approved the manuscript. NT, HC, AL, and JP provided consultative advice and approved the final manuscript.

## Funding

HH, DH, DK, AL, and HC are members of COST Action (TINNET BM1306) a research program funded under the Biomedicine and Molecular Biosciences European Cooperation in Science and Technology (COST) Action framework. Travel, subsistence, and accommodation for them to participate in Tinnet meetings has been funded by Tinnet and that has been an opportunity to enhance networking collaboration between them. HH has received a Ph.D. Grant from Jmellosaude (20,000€). DH is funded by the National Institute for Health Research (NIHR) Biomedical Research Unit programme. The views expressed are those of the authors and not the funder.

### Conflict of interest statement

The authors declare that the research was conducted in the absence of any commercial or financial relationships that could be construed as a potential conflict of interest.
