# Supplementary material for: Pathophysiology, Diagnosis and Treatment of Somatosensory Tinnitus: A Scoping Review
Source: Front Neurosci. 2017 Apr 28;11:207. doi: 10.3389/fnins.2017.00207 (PMC5408030; doi:10.3389/fnins.2017.00207)
Supplement: Supplementary file 1 [file DataSheet1.docx]

Supplementary Material

Pathophysiology, diagnosis and treatment of somatosensory tinnitus; a scoping review

Haúla Haider*, Derek J Hoare, Raquel Costa, Iskra Potgieter, Dimitris Kikidis, Alec Lapira, Christos Nikitas, Helena Caria, Nuno Trigueiros, João Paço.

* Correspondence: Corresponding Author: [hfhaider@gmail.com](mailto:hfhaider@gmail.com)

**Appendix 1. Example search PubMed**

(somato[All Fields] OR somatoacouphenes[All Fields] OR somatoacoustic[All Fields] OR somatoafferent[All Fields] OR somatoagnosia[All Fields] OR somatoalgetic[All Fields] OR somatoalgia[All Fields] OR somatoalgic[All Fields] OR somatoanal[All Fields] OR somatoanalysis[All Fields] OR somatoarcuate[All Fields] OR somatoatrophs[All Fields] OR somatoautonomic[All Fields] OR somatoaxon[All Fields] OR somatoaxonal[All Fields] OR somatobiological[All Fields] OR somatobiologicos[All Fields] OR somatobladder[All Fields] OR somatoblast[All Fields] OR somatoblasten[All Fields] OR somatoblasts[All Fields] OR somatocardiac[All Fields] OR somatocardiovascular[All Fields] OR somatocarta[All Fields] OR somatocentered[All Fields] OR somatocentesis[All Fields] OR somatocentric[All Fields] OR somatoceptive[All Fields] OR somatochart[All Fields] OR somatocharts[All Fields] OR somatocheskikh[All Fields] OR somatochlora[All Fields] OR somatocism[All Fields] OR somatoclone[All Fields] OR somatocnen[All Fields] OR somatocoel[All Fields] OR somatocoelic[All Fields] OR somatocoels[All Fields] OR somatocognitive[All Fields] OR somatocor[All Fields] OR somatocorticotrophic[All Fields] OR somatocrinin[All Fields] OR somatocrinine[All Fields] OR somatocrininergic[All Fields] OR somatocyte[All Fields] OR somatocytes[All Fields] OR somatodendretic[All Fields] OR somatodendric[All Fields] OR somatodendritc[All Fields] OR somatodendrite[All Fields] OR somatodendrites[All Fields] OR somatodendritic[All Fields] OR somatodendritically[All Fields] OR somatodendritnye[All Fields] OR somatodenritic[All Fields] OR somatodentritic[All Fields] OR somatodiagnostic[All Fields] OR somatodiagnostics[All Fields] OR somatodysgnosia[All Fields] OR somatodyspraxia[All Fields] OR somatoectoderm[All Fields] OR somatoefferents[All Fields] OR somatoencephalic[All Fields] OR somatoendocrine[All Fields] OR somatoendokrinnye[All Fields] OR somatoendokrinnykh[All Fields] OR somatoensory[All Fields] OR somatoerogenic[All Fields] OR somatoestesici[All Fields] OR somatofalk[All Fields] OR somatofisiologica[All Fields] OR somatofom[All Fields] OR somatofomer[All Fields] OR somatoform[All Fields] OR somatoform'[All Fields] OR somatoformal[All Fields] OR somatoformand[All Fields] OR somatoforme[All Fields] OR somatoformed[All Fields] OR somatoformem[All Fields] OR somatoformen[All Fields] OR somatoformer[All Fields] OR somatoformes[All Fields] OR somatoformi[All Fields] OR somatoformic[All Fields] OR somatoformiset[All Fields] OR somatoformnoi[All Fields] OR somatoformnykh[All Fields] OR somatoformnymi[All Fields] OR somatoforms[All Fields] OR somatofornykh[All Fields] OR somatofugal[All Fields] OR somatofugally[All Fields] OR somatofunction[All Fields] OR somatofungal[All Fields] OR somatofunktsional'nyi[All Fields] OR somatogamous[All Fields] OR somatogastric[All Fields] OR somatogastrici[All Fields] OR somatogen[All Fields] OR somatogenc[All Fields] OR somatogene[All Fields] OR somatogenen[All Fields] OR somatogeneous[All Fields] OR somatogener[All Fields] OR somatogenes[All Fields] OR somatogenese[All Fields] OR somatogenesis[All Fields] OR somatogenetic[All Fields] OR somatogenetics[All Fields] OR somatogenia[All Fields] OR somatogenias[All Fields] OR somatogenic[All Fields] OR somatogenic'[All Fields] OR somatogenically[All Fields] OR somatogenies[All Fields] OR somatogenii[All Fields] OR somatogenital[All Fields] OR somatogenitale[All Fields] OR somatogenna[All Fields] OR somatogenno[All Fields] OR somatogennogo[All Fields] OR somatogennoi[All Fields] OR somatogennych[All Fields] OR somatogennye[All Fields] OR somatogennyi[All Fields] OR somatogennykh[All Fields] OR somatogennym[All Fields] OR somatogenotoxicity[All Fields] OR somatogenous[All Fields] OR somatogens[All Fields] OR somatogeriatric[All Fields] OR somatognathic[All Fields] OR somatognathous[All Fields] OR somatognosia[All Fields] OR somatognosic[All Fields] OR somatognosically[All Fields] OR somatognosici[All Fields] OR somatognosie[All Fields] OR somatognosique[All Fields] OR somatognosiques[All Fields] OR somatognosis[All Fields] OR somatognostic[All Fields] OR somatognozie[All Fields] OR somatognozije[All Fields] OR somatogonadotrope[All Fields] OR somatogonadotropes[All Fields] OR somatogram[All Fields] OR somatograma[All Fields] OR somatogramm[All Fields] OR somatogramme[All Fields] OR somatogramms[All Fields] OR somatogrammy[All Fields] OR somatogramography[All Fields] OR somatograms[All Fields] OR somatographic[All Fields] OR somatography[All Fields] OR somatogravic[All Fields] OR somatogravic'[All Fields] OR somatogropo[All Fields] OR somatogyral[All Fields] OR somatohaploid[All Fields] OR somatohaploids[All Fields] OR somatohennii[All Fields] OR somatohorm[All Fields] OR somatohormone[All Fields] OR somatoichni[All Fields] OR somatoirope[All Fields] OR somatokine[All Fields] OR somatokinetic[All Fields] OR somatokryniny[All Fields] OR somatolactin[All Fields] OR somatolactins[All Fields] OR somatolaction[All Fields] OR somatolactogen[All Fields] OR somatolactogenic[All Fields] OR somatolactogens[All Fields] OR somatolactotrope[All Fields] OR somatolactotropes[All Fields] OR somatolactotroph[All Fields] OR somatolactotrophes[All Fields] OR somatolactotrophic[All Fields] OR somatolactotrophinomas[All Fields] OR somatolactotrophs[All Fields] OR somatolactotropic[All Fields] OR somatolactotropinoma[All Fields] OR somatolateral[All Fields] OR somatoliberin[All Fields] OR somatoliberine[All Fields] OR somatoliberinoma[All Fields] OR somatoliberinomas[All Fields] OR somatoliberinome[All Fields] OR somatoline[All Fields] OR somatolocomotor[All Fields] OR somatologia[All Fields] OR somatologia'[All Fields] OR somatologic[All Fields] OR somatologic'os[All Fields] OR somatological[All Fields] OR somatologicheskaia[All Fields] OR somatologicos[All Fields] OR somatologie[All Fields] OR somatologische[All Fields] OR somatologischen[All Fields] OR somatologism[All Fields] OR somatologues[All Fields] OR somatology[All Fields] OR somatolysis[All Fields] OR somatolytic[All Fields] OR somatom[All Fields] OR somatom25[All Fields] OR somatomadin[All Fields] OR somatomam[All Fields] OR somatomamatrophin[All Fields] OR somatomammatropin[All Fields] OR somatomammocorticotrophic[All Fields] OR somatomammogenic[All Fields] OR somatomammothophs[All Fields] OR somatomammotrofin[All Fields] OR somatomammotrope[All Fields] OR somatomammotropes[All Fields] OR somatomammotroph[All Fields] OR somatomammotrophes[All Fields] OR somatomammotrophic[All Fields] OR somatomammotrophin[All Fields] OR somatomammotrophine[All Fields] OR somatomammotrophique[All Fields] OR somatomammotrophs[All Fields] OR somatomammotropic[All Fields] OR somatomammotropin[All Fields] OR somatomammotropina[All Fields] OR somatomammotropine[All Fields] OR somatomammotropinico[All Fields] OR somatomammotropinoma[All Fields] OR somatomammotropins[All Fields] OR somatomammotropiny[All Fields] OR somatomammotropnogo[All Fields] OR somatomamotrofina[All Fields] OR somatomamotrophic[All Fields] OR somatomamotropico[All Fields] OR somatomamotropin[All Fields] OR somatomamotropina[All Fields] OR somatome[All Fields] OR somatomedia[All Fields] OR somatomedian[All Fields] OR somatomedians[All Fields] OR somatomedin[All Fields] OR somatomedin'[All Fields] OR somatomedin'hypothesis[All Fields] OR somatomedin's[All Fields] OR somatomedina[All Fields] OR somatomedinaktivitat[All Fields] OR somatomedinas[All Fields] OR somatomedine[All Fields] OR somatomedinemia[All Fields] OR somatomediner[All Fields] OR somatomedines[All Fields] OR somatomeding[All Fields] OR somatomedinic[All Fields] OR somatomedinica[All Fields] OR somatomedinlike[All Fields] OR somatomedinovaia[All Fields] OR somatomedinove[All Fields] OR somatomedinovej[All Fields] OR somatomedinovoi[All Fields] OR somatomedinovuiu[All Fields] OR somatomedins[All Fields] OR somatomedins'[All Fields] OR somatomedinu[All Fields] OR somatomedizinischen[All Fields] OR somatomedogenic[All Fields] OR somatomedrin[All Fields] OR somatomedyn[All Fields] OR somatomedyna[All Fields] OR somatomedynowej[All Fields] OR somatomedyny[All Fields] OR somatomegaly[All Fields] OR somatomental[All Fields] OR somatomentalis[All Fields] OR somatomerical[All Fields] OR somatomerin[All Fields] OR somatomesodermal[All Fields] OR somatometer[All Fields] OR somatometria[All Fields] OR somatometriaa[All Fields] OR somatometric[All Fields] OR somatometrica[All Fields] OR somatometrical[All Fields] OR somatometrically[All Fields] OR somatometricas[All Fields] OR somatometricheskaia[All Fields] OR somatometricheskie[All Fields] OR somatometricheskikh[All Fields] OR somatometricheskogo[All Fields] OR somatometrici[All Fields] OR somatometrick'e[All Fields] OR somatometricka[All Fields] OR somatometricke[All Fields] OR somatometrickeho[All Fields] OR somatometricky[All Fields] OR somatometrickych[All Fields] OR somatometrickym[All Fields] OR somatometrickymi[All Fields] OR somatometrico[All Fields] OR somatometricos[All Fields] OR somatometrics[All Fields] OR somatometrie[All Fields] OR somatometriques[All Fields] OR somatometrisch[All Fields] OR somatometrische[All Fields] OR somatometrischen[All Fields] OR somatometrischer[All Fields] OR somatometry[All Fields] OR somatomicronuclei[All Fields] OR somatommammotropin[All Fields] OR somatommammptropin[All Fields] OR somatommamotroph[All Fields] OR somatommamotropin[All Fields] OR somatommamotropins[All Fields] OR somatommaotropic[All Fields] OR somatommatrophique[All Fields] OR somatommatropin[All Fields] OR somatomommotropic[All Fields] OR somatomoral[All Fields] OR somatomorfo[All Fields] OR somatomorfos[All Fields] OR somatomorphe[All Fields] OR somatomorphes[All Fields] OR somatomorphic[All Fields] OR somatomorphism[All Fields] OR somatomorphous[All Fields] OR somatomorphs[All Fields] OR somatomoteur[All Fields] OR somatomotion[All Fields] OR somatomotor[All Fields] OR somatomotor'[All Fields] OR somatomotoras[All Fields] OR somatomotori[All Fields] OR somatomotoric[All Fields] OR somatomotoricku[All Fields] OR somatomotorie[All Fields] OR somatomotorio[All Fields] OR somatomotorischer[All Fields] OR somatomotornoe[All Fields] OR somatomotornogo[All Fields] OR somatomotornykh[All Fields] OR somatomu[All Fields] OR somatomutagenic[All Fields] OR somatomy[All Fields] OR somatomy'[All Fields] OR somaton[All Fields] OR somatonergic[All Fields] OR somatoneurologic[All Fields] OR somatoneurological[All Fields] OR somatoneurology[All Fields] OR somatoneurons[All Fields] OR somatoneurosen[All Fields] OR somatoneuroses[All Fields] OR somatoneurotic[All Fields] OR somatoneurotischen[All Fields] OR somatonevrologicheskie[All Fields] OR somatonevrologycheskoj[All Fields] OR somatonevrologycheskuju[All Fields] OR somatonorm[All Fields] OR somatonuclear[All Fields] OR somatopancreatic[All Fields] OR somatoparaphrenia[All Fields] OR somatoparaphrenic[All Fields] OR somatoparaphrenie[All Fields] OR somatoparasympathetic[All Fields] OR somatoparietal[All Fields] OR somatopathic[All Fields] OR somatopathies[All Fields] OR somatopause[All Fields] OR somatopause'[All Fields] OR somatopauza[All Fields] OR somatopenia[All Fields] OR somatoperception[All Fields] OR somatoperceptive[All Fields] OR somatoperceptual[All Fields] OR somatopetal[All Fields] OR somatopetally[All Fields] OR somatophoric[All Fields] OR somatophorme[All Fields] OR somatophormic[All Fields] OR somatophysical[All Fields] OR somatophysiological[All Fields] OR somatophysiologycal[All Fields] OR somatophysiometric[All Fields] OR somatophysiometrischen[All Fields] OR somatopia[All Fields] OR somatopic[All Fields] OR somatopically[All Fields] OR somatopicly[All Fields] OR somatoplasm[All Fields] OR somatoplastic[All Fields] OR somatoplegia[All Fields] OR somatoplegia'[All Fields] OR somatoplerual[All Fields] OR somatopleur[All Fields] OR somatopleura[All Fields] OR somatopleurae[All Fields] OR somatopleural[All Fields] OR somatopleure[All Fields] OR somatopleure's[All Fields] OR somatopleuric[All Fields] OR somatoplot[All Fields] OR somatoplots[All Fields] OR somatopolovogo[All Fields] OR somatopraxic[All Fields] OR somatopraxis[All Fields] OR somatopressor[All Fields] OR somatoprim[All Fields] OR somatoprolactinic[All Fields] OR somatoprolactinoma[All Fields] OR somatoprolactinomas[All Fields] OR somatoprostheses[All Fields] OR somatoprosthesis[All Fields] OR somatoprosthetic[All Fields] OR somatoprotezy[All Fields] OR somatoproximal[All Fields] OR somatopsichica[All Fields] OR somatopsichiche[All Fields] OR somatopsichici[All Fields] OR somatopsichico[All Fields] OR somatopsikhiatricheskikh[All Fields] OR somatopsikhiatrii[All Fields] OR somatopsikhicheski[All Fields] OR somatopsikhicheskie[All Fields] OR somatopsikhicheskii[All Fields] OR somatopsikhicheskikh[All Fields] OR somatopsikhicheskogo[All Fields] OR somatopsikhovegetativnye[All Fields] OR somatopsiquica[All Fields] OR somatopstatin[All Fields] OR somatopsyche[All Fields] OR somatopsychiatric[All Fields] OR somatopsychiatrics[All Fields] OR somatopsychiatry[All Fields] OR somatopsychiaues[All Fields] OR somatopsychic[All Fields] OR somatopsychic'[All Fields] OR somatopsychical[All Fields] OR somatopsychics[All Fields] OR somatopsychiczna[All Fields] OR somatopsychiczne[All Fields] OR somatopsychicznej[All Fields] OR somatopsychiek[All Fields] OR somatopsychik[All Fields] OR somatopsychique[All Fields] OR somatopsychis[All Fields] OR somatopsychisch[All Fields] OR somatopsychische[All Fields] OR somatopsychischen[All Fields] OR somatopsychischer[All Fields] OR somatopsychisches[All Fields] OR somatopsychologic[All Fields] OR somatopsychological[All Fields] OR somatopsychologie[All Fields] OR somatopsychology[All Fields] OR somatopsychosis[All Fields] OR somatopsychosocial[All Fields] OR somatopsychotherapeutic[All Fields] OR somatopsychotherapy[All Fields] OR somatopsychotic[All Fields] OR somatopsykiske[All Fields] OR somatoptype[All Fields] OR somatopy[All Fields] OR somatoreactive[All Fields] OR somatoreception[All Fields] OR somatoreceptor[All Fields] OR somatorecipient[All Fields] OR somatorelin[All Fields] OR somatorenal[All Fields] OR somatorepresentation[All Fields] OR somatorform[All Fields] OR somatoria[All Fields] OR somatorias[All Fields] OR somatorio[All Fields] OR somatormone[All Fields] OR somatorophic[All Fields] OR somatoropin[All Fields] OR somatoropnaia[All Fields] OR somatory[All Fields] OR somatorype[All Fields] OR somatos[All Fields] OR somatosatatin[All Fields] OR somatosatin[All Fields] OR somatosch[All Fields] OR somatoschisis[All Fields] OR somatoscintigraphy[All Fields] OR somatoscopic[All Fields] OR somatoscopically[All Fields] OR somatoscopy[All Fields] OR somatose[All Fields] OR somatosecretagogue[All Fields] OR somatosehypothese[All Fields] OR somatosemsory[All Fields] OR somatosenasory[All Fields] OR somatosenescence[All Fields] OR somatosenoriali[All Fields] OR somatosenory[All Fields] OR somatosenroy[All Fields] OR somatosens[All Fields] OR somatosensary[All Fields] OR somatosensation[All Fields] OR somatosensations[All Fields] OR somatosensatory[All Fields] OR somatosense[All Fields] OR somatosensensory[All Fields] OR somatosensibel[All Fields] OR somatosensible[All Fields] OR somatosensiblen[All Fields] OR somatosensibler[All Fields] OR somatosensibles[All Fields] OR somatosensing[All Fields] OR somatosensitivas[All Fields] OR somatosensitive[All Fields] OR somatosensitivity[All Fields] OR somatosensitivo[All Fields] OR somatosensitivos[All Fields] OR somatosensitve[All Fields] OR somatosensor[All Fields] OR somatosensori[All Fields] OR somatosensoria[All Fields] OR somatosensorial[All Fields] OR somatosensoriale[All Fields] OR somatosensoriales[All Fields] OR somatosensoriali[All Fields] OR somatosensoriality[All Fields] OR somatosensorially[All Fields] OR somatosensoric[All Fields] OR somatosensorical[All Fields] OR somatosensorically[All Fields] OR somatosensorickych[All Fields] OR somatosensorieblock[All Fields] OR somatosensoriel[All Fields] OR somatosensorielle[All Fields] OR somatosensorielles[All Fields] OR somatosensoriels[All Fields] OR somatosensorily[All Fields] OR somatosensorimotor[All Fields] OR somatosensorisch[All Fields] OR somatosensorische[All Fields] OR somatosensorischen[All Fields] OR somatosensorischer[All Fields] OR somatosensoriyal[All Fields] OR somatosensoriyel[All Fields] OR somatosensornoe[All Fields] OR somatosensornogo[All Fields] OR somatosensornoi[All Fields] OR somatosensornuiu[All Fields] OR somatosensornye[All Fields] OR somatosensornyi[All Fields] OR somatosensornykh[All Fields] OR somatosensornym[All Fields] OR somatosensornymi[All Fields] OR somatosensors[All Fields] OR somatosensory[All Fields] OR somatosensory'[All Fields] OR somatosensoryczne[All Fields] OR somatosensoryczny[All Fields] OR somatosensorycznych[All Fields] OR somatosensorynykh[All Fields] OR somatosensotnykh[All Fields] OR somatosensoty[All Fields] OR somatosenstory[All Fields] OR somatosentory[All Fields] OR somatosenzitivni[All Fields] OR somatosenzitivnih[All Fields] OR somatosenzoricka[All Fields] OR somatosenzoricke[All Fields] OR somatosenzorickeho[All Fields] OR somatosenzoricki[All Fields] OR somatosenzorickog[All Fields] OR somatosenzoricky[All Fields] OR somatosenzoricnih[All Fields] OR somatosenzornaia[All Fields] OR somatosenzorne[All Fields] OR somatosenzorni[All Fields] OR somatosenzornih[All Fields] OR somatosenzornoi[All Fields] OR somatosenzornykh[All Fields] OR somatosenzorskih[All Fields] OR somatosesensory[All Fields] OR somatosetnsory[All Fields] OR somatosexual[All Fields] OR somatosexually[All Fields] OR somatosexualni[All Fields] OR somatosexualniho[All Fields] OR somatosexuellen[All Fields] OR somatosimpaticheskie[All Fields] OR somatosimpaticheskii[All Fields] OR somatosimpaticheskogo[All Fields] OR somatosin[All Fields] OR somatosis[All Fields] OR somatosization[All Fields] OR somatosized[All Fields] OR somatoskopicheskikh[All Fields] OR somatoskopicka[All Fields]) AND ("tinnitus"[MeSH Terms] OR "tinnitus"[All Fields])
