# Supplementary material for: Pathophysiology, Diagnosis and Treatment of Somatosensory Tinnitus: A Scoping Review
Source: Front Neurosci. 2017 Apr 28;11:207. doi: 10.3389/fnins.2017.00207 (PMC5408030; doi:10.3389/fnins.2017.00207)
Supplement: Supplementary file 2 [file DataSheet2.docx]

***Supplementary Material***

Pathophysiology, diagnosis and treatment of somatosensory tinnitus; a scoping review

Haúla Haider*, Derek J Hoare, Raquel Costa, Iskra Potgieter, Dimitris Kikidis, Alec Lapira, Christos Nikitas, Helena Caria, Nuno Trigueiros, João Paço.

* Correspondence: Corresponding Author: [hfhaider@gmail.com](mailto:hfhaider@gmail.com)

**Appendix 2. Summary of studies about etiology of somatosensory tinnitus**

| **Author** | **N** | **Hypothesis/aim** | **Methodology** | **Results/Conclusion** |
| --- | --- | --- | --- | --- |
| Koehler and Shore, 2013 | 16 female pigmented guinea pigs | "The stimulus timing dependence of bimodal plasticity in a tinnitus model" | Noise exposure induced to the subjects; auditory brainstem measure; acute DCN recording preparation | "Alterations in DCN bimodal spike timing-dependent plasticity as underlying mechanisms in tinnitus, opening the way for a therapeutic target." |
| Rocha et al., 2008 | 94+94 | Report the association between tinnitus and myofascial trigger points, if tinnitus is ipsilateral to the trigger points and the modulation abilities in these patients | Evaluation protocol and digital pressure | Myofascial trigger points are common among tinnitus patients and pressure may modulate tinnitus |
| Sanchez et al., 2007 | 38 | assess the reliability of tinnitus modulation and observe the effects of prolonged muscle contractions repetition | 9 manoeuvres in test and retest situations | "Repetition of manoeuvres for 2 months altered the pattern of modulation" |
| Levine et al., 2003 | 62 | assess the effect of somatic contraction in 1) non-clinical population; 2) profoundly deaf | questionnaire and somatic testing of 25 manoeuvres | "Somatic modulation of tinnitus demonstrates the interaction of the central auditory nervous system with the central somatosensory system, |
| Simmons et al., 2008 | 113 (eye modulation), 45 (other manoeuvres) | assess the somatic modulation of tinnitus loudness | questionnaire, eye movements, performance of 42 manoeuvres | "The high prevalence of somatic tinnitus serves to illustrate the complex multimodal interactions that exist between the auditory pathway and other sensory-motor systems innervating the head, neck, shoulders, and eyes." |
| Lockwood et al., 2001 | 8+7 | "changes in the neural activity associated with GET would be identified by changes in cerebral blood flow (CBF) using PET" | GET and PET to map the neural sites activated by lateral gaze | "tinnitus and its related phenomena are the result of plastic changes in the central auditory system and the formation of anomalous links between the central auditory pathways and other neural elements" |
| Reyes et al., 2002 | 10 | reduction in tinnitus loudness in the tinnitus group and no effect in the control group | audiologic evaluation, imaging protocol | "tinnitus originates in the central auditory system rather than the cochlea." |
| Lockwood et al., 1998 | 10 | changes in the loudness of the tinnitus are related to changes in CBF, which can be mapped through CBF with PET. | cerebral blood flow through PET during stimulation with pure tones; audiometry and positron emission tomography | "neural systems mediating tinnitus may be linked to systems controlling emotions via the hippocampus, a portion of the limbic system, that is the gateway to centres mediating emotional control and an important component of memory systems! |
| Chole and Parker, 1992 | 338 patients and 326 controls | determine if tinnitus and vertigo are more prevalent in TMD patients than in age-matched controls | questionnaire | "Tinnitus and vertigo symptoms were significantly more prevalent in the TMD group than in either of the control groups. The mechanism" |
| Vielsmeier et al., 2012 | 1204 | history of TMJ complaints alternate tinnitus phenotype | TRI database search (1204 patients) | 22% TMJ positive (patients) were younger, had an earlier tinnitus onset. Significantly difference in masking effectiveness. |
| Bernhardt et al., 2011 | 3300 | signs and symptoms of TMD can be identified as risk factors for tinnitus | questionnaire, medical examination, oral health examination, health-related interview | Signs of TMD are a risk factor for the development of tinnitus |
| Vielsmeier et al., 2011 | 30 patients with TMD and 61 without | TMJ disorder is the cause of tinnitus? Is tinnitus a symptom of TMJ? Tinnitus-TMJ is different from tinnitus dissociated with TMJ | clinical examination, questionnaires (Tinnitus Sample Case History, Tinnitus Handicap Inventory) | "Classical risk factors for tinnitus (age, male gender, hearing loss) are less relevant in tinnitus patients with TMJ disorder, suggesting a causal role of TMJ pathology in the generation and maintenance of tinnitus" |
| Burgers et al., 2013 | 25 | Assessment of comorbidity of tinnitus and TMD; effect of TMD therapy on tinnitus | participants received a customised dental functional therapy; assessment at baseline, 3 months, 5 months follow -up | TMD therapy improved symptoms in 11/25 (44%) of participants. significant correlation of tinnitus and TMD; positive effect of treatment in tinnitus |
| Osterman et al., 2016 | 70 | sound enrichment and white noise stimulation might decrease tinnitus and associated somatic symptoms | TRT protocol, Jastreboff Structured Interview, Tinnitus Handicap Inventory | "Somatic tinnitus is a frequent and underestimated.TRT significantly improved tinnitus and accompanying facial dysesthesia |
| Wright and Bifano, 1997b | 40 | investigation of the improvement of tinnitus after TMD treatment | evaluation of improvement of symptoms; correlation of tinnitus change and participants characteristics | 21/40 participants resolve tinnitus; 12/40 report improvement; 7/40 had unchanged tinnitus; no report for aggravation of symptoms; tinnitus significantly associate with age, severity, stress, comorbidity with TMD, bruxism |
