# Supplementary material for: Pathophysiology, Diagnosis and Treatment of Somatosensory Tinnitus: A Scoping Review
Source: Front Neurosci. 2017 Apr 28;11:207. doi: 10.3389/fnins.2017.00207 (PMC5408030; doi:10.3389/fnins.2017.00207)
Supplement: Supplementary file 3 [file DataSheet3.docx]

***Supplementary Material***

Pathophysiology, diagnosis and treatment of somatosensory tinnitus; a scoping review

Haúla Haider*, Derek J Hoare, Raquel Costa, Iskra Potgieter, Dimitris Kikidis, Alec Lapira, Christos Nikitas, Helena Caria, Nuno Trigueiros, João Paço.

* Correspondence: Corresponding Author: [hfhaider@gmail.com](mailto:hfhaider@gmail.com)

**Appendix 3. Summary of studies about epidemiology of somatosensory tinnitus**

| **Author** | **N** | **Hypothesis/aim** | **Methodology** | **Results/conclusion** |
| --- | --- | --- | --- | --- |
| Ward et al., 2015 | 608 | assess somatic tinnitus trait within a large UK cohort using an unselected sample. | meta-analysis used randomised data that had been collected as part of various research studies led by the National Institute for Health Research (NIHR) | "The prevalence of self-reported ability to modulate tinnitus through somatic manoeuvres was 16% in a general cohort of people with tinnitus. We define this as somatic tinnitus. Somatic tinnitus was significantly predicted by age, pulsatility, loudness and TMJ complaints" |
| Burgers et al., 2013 | 25 | Assessment of comorbidity of tinnitus and TMD; effect of TMD therapy on tinnitus | participants received a customised dental functional therapy; assessment at baseline, 3 months, 5 months follow -up | TMD therapy improved symptoms in 11/25 (44%) of participants. significant correlation of tinnitus and TMD; positive effect of treatment in tinnitus |
| Wright and Bifano, 1997a | 93 | Comorbidity of TMD and tinnitus | evaluation of tinnitus improvement after a TMD treatment | history and clinical test help identify the coexistence of tinnitus and TMD; tinnitus improvement after TMD treatment |
| Vielsmeier et al., 2011 | 30 patients with TMD and 61 without | TMJ disorder is the cause of tinnitus? Is tinnitus a symptom of TMJ? Tinnitus-TMJ is different from tinnitus dissociated with TMJ | clinical examination, questionnaires (Tinnitus Sample Case History, Tinnitus Handicap Inventory) | "Classical risk factors for tinnitus (age, male gender, hearing loss) are less relevant in tinnitus patients with TMJ disorder, suggesting a causal role of TMJ pathology in the generation and maintenance of tinnitus. Based on this finding, treatment of TMJ disorder may represent a causally oriented treatment strategy for tinnitus." |
| Vielsmeier et al., 2012 | 1204 | history of TMJ complaints alternate tinnitus phenotype | TRI database search (1204 patients) | 22% TMJ positive (patients) were younger, had an earlier tinnitus onset. Significantly difference in masking effectiveness. |
| Abel and Levine, 2004 | 60 patients and 60 controls | "the ability of nonclinical subjects to modulate their phantom sound perception is less than that of the Massachusetts Eye and Ear Infirmary Tinnitus clinic patient pool." | questionnaire, performance of 25 muscle contractions | "Somatic influences upon auditory perception are not limited to tinnitus sufferers but appear to be a fundamental property of the auditory system." |
| Won et al., 2013 | 88 male and 75 female | "examine the relationship between the characteristics of patients with tinnitus and their response to somatic testing to identify the factors associated with successful somatic modulation" | 19 neck and jaw manoeuvres, physiological and audiological profile | "Treatment modalities involving the somatosensory systems, and particularly manual therapy, should now be re-assessed in the subgroup of patients with cervicogenic somatosensory tinnitus." |
| Coles, 1984 | 522+ 5000+ 8069+7645 (phase); OPCS: 23000 | integrate the both two large scale surveys (National Study of Hearing and the General Household Survey) | postal questionnaire (NSH); personal interview (OPCS) | 0.5% of patients reported a significant decrease in life quality due to tinnitus, and 4% indicate a moderate degree of annoyance; 4 millions adults suffer from this disorder in the UK. |
| Schaette and McApline, 2011 | 15 tinnitus; 18 controls | deafferentation of NA fibbers due to temporary hearing loss may trigger the development of neural correlate of tinnitus in central structure | audiograms; auditory brainstem responses evaluated (Medelec Synergy T-EP system | tinnitus could arise from a homeostatic response of neurons in the central auditory system to reduced auditory nerve input in the absence of elevated hearing thresholds. |
| Shargorodsky et al., 2010 | 14178 | the relations between tinnitus and other demographic and health factors are minimally characterized in the current literature | interview and patient examination | "The prevalence of frequent tinnitus is highest among older adults, non-Hispanic whites, former smokers, and adults with hypertension, hearing impairment, loud noise exposure, or generalized anxiety disorder". |
| Coelho et al., 2007 | 258 males and 229 females | estimate the prevalence of tinnitus and explore the risk factors in school-aged children age 5–12 years. | questionnaire data from parents and interviews with children | Tinnitus is common among children. "Appropriated instruments to evaluate tinnitus annoyance in childhood still have to be validated and are necessary to classify the degree of distressing of this symptom among children to determine possible therapeutic effects of intervention and maturation in this population." |
| Chole and Parker, 1992 | 338 patients and 326 controls | determine if tinnitus and vertigo are more prevalent in TMD patients than in age-matched controls | questionnaire | "Tinnitus and vertigo symptoms were significantly more prevalent in the TMD group than in either of the control groups. The mechanism" |
