# Supplementary material for: Pathophysiology, Diagnosis and Treatment of Somatosensory Tinnitus: A Scoping Review
Source: Front Neurosci. 2017 Apr 28;11:207. doi: 10.3389/fnins.2017.00207 (PMC5408030; doi:10.3389/fnins.2017.00207)
Supplement: Supplementary file 4 [file DataSheet4.docx]

***Supplementary Material***

Pathophysiology, diagnosis and treatment of somatosensory tinnitus; a scoping review

Haúla Haider*, Derek J Hoare, Raquel Costa, Iskra Potgieter, Dimitris Kikidis, Alec Lapira, Christos Nikitas, Helena Caria, Nuno Trigueiros, João Paço.

* Correspondence: Corresponding Author: [hfhaider@gmail.com](mailto:hfhaider@gmail.com)

**Appendix 4. Summary of studies related to diagnosis of somatosensory tinnitus**

| **Author** | **Hypothesis/ aim** | **Methodology** | **Results/Conclusion** |
| --- | --- | --- | --- |
| Levine et al., 1999 | Tinnitus can be modulate through manoeuvres | identify characteristics of tinnitus associated with somatic events | 68% of 70 patients modulate tinnitus through manoeuvres |
| Sanchez et al., 2002 | Set of 16 manoeuvres | Tinnitus patients vs. healthy subjects | specific manoeuvres modulate tinnitus; significant difference between groups |
| Abel and Levine, 2004 | modulate tinnitus with head and neck contractions | elicitation of tinnitus-like auditory perception with head-neck contractions in non clinical subjects | 80% of non clinical subjects could modulate tinnitus |
| Vielsmeier et al., 2012 | history of TMJ complaints alternate tinnitus phenotype | TRI database search (1204 patients) | 22% TMJ positive (patients) were younger, had an earlier tinnitus onset. Significantly difference in masking effectiveness. |
| Lookwood et al, 2001 | Tinnitus could be modulated by gaze | PET and MRI scan in patients with gaze evoked tinnitus developed after acoustic neuroma removal | Patients with Gaze-evoked/modulate tinnitus have plastic changes in multiple neural systems |
| Simons et al., 2008 | Tinnitus modulations by jaw clench | Performace of jaw clench in tinnitus patients | 90% increase of loudness, 50% pitch intensity |
| Won et al., 2013 | Tinnitus modulation through neck and jaw manoeuvres | Implementation of manoeuvres in tinnitus patients | 57% able to modulate tinnitus loudness (decrease or increase) |
