# Supplementary material for: Pathophysiology, Diagnosis and Treatment of Somatosensory Tinnitus: A Scoping Review
Source: Front Neurosci. 2017 Apr 28;11:207. doi: 10.3389/fnins.2017.00207 (PMC5408030; doi:10.3389/fnins.2017.00207)
Supplement: Supplementary file 5 [file DataSheet5.docx]

***Supplementary Material***

Pathophysiology, diagnosis and treatment of somatosensory tinnitus; a scoping review

Haúla Haider*, Derek J Hoare, Raquel Costa, Iskra Potgieter, Dimitris Kikidis, Alec Lapira, Christos Nikitas, Helena Caria, Nuno Trigueiros, João Paço.

* Correspondence: Corresponding Author: [hfhaider@gmail.com](mailto:hfhaider@gmail.com)

**Appendix 5. Summary of studies about somatosensory tinnitus treatment**

| **Author** | **N** | **Hypothesis/aim** | **Methodology** | **Results/ Conclusion** |
| --- | --- | --- | --- | --- |
| Attanasio G., et al,2015 | 55/86 participants (18-60 y.o) devided in 3 groups | Correlation between tinnitus and TMJ dysfunction | 3 study groups; all received a occlusal splint for 6 months; pre and post treatment severity of symptoms using VAS scale and THI questionnaire | Statistically significant decrease of VAS and THI. Positive effect of occlusal splint therapy in TMJ dysfunction. |
| Tullberg M., et al,2006 | 120 participants; 50 participants in waiting list (control group) | prevalence of TMD disorders and tinnitus; effect of TMD treatment on tinnitus | 120 participants underwent clinical examination of the masticatory system; 2 groups (intervention group = 73/96 with TMD; control group =50); 2 years follow - up | 80% signs of TMD during examination; 43% report improvement of tinnitus (2 years follow -up); 12% report improvement in the waiting list; statistically significant difference between groups (p<0.001). |
| Wright & Biffano, 1997a | 93 participants | Comorbidity of TMD and tinnitus | evaluation of tinnitus improvement after a TMD treatment | Tinnitus improvement after treatment; history and clinical test help identify the coexistence of tinnitus and TMD; tinnitus improvement after TMD treatment |
| Wright & Biffano, 1997b | 40 participants | investigation of the improvement of tinnitus after TMD treatment | evaluation of improvement of symptoms; correlation of tinnitus change and participants characteristics | 21/40 participants resolve tinnitus; 12/40 report improvement; 7/40 had unchanged tinnitus; no report for aggravation of symptoms; tinnitus significantly associate with age, severity, stress, comorbidity with TMD, bruxism |
| Wright, 2000 |  | TMD treatment improves otologic symptoms | Participants provided with a dental orthotic and with self care instructions; evaluation at baseline, 3 months and 6 months follow -up | 64% improvement of tinnitus,91% of dizziness,87% of otalgia and 92% of TMD |
| Rubinstein & Erlandsson,1991 | 42 participants | stomatognathic analysis of tinnitus and craniomandibular disorders (CMD) | participants had a history taken, a stomatognathic examination and ask to keep a diary of tinnitus symptoms for 2 weeks | Bruxism and jaw tenderness are related to fluctuating tinnitus |
| Buergers R., et al, 2013 | 25/951 patients with tinnitus and TMD; no control group | Assessment of comorbidity of tinnitus and TMD; effect of TMD therapy on tinnitus | participants received a customised dental functional therapy; assessment at baseline, 3 months, 5 months follow -up | TMD therapy improved symptoms in 11/25 (44%) of participants. significant correlation of tinnitus and TMD; positive effect of treatment in tinnitus |
| Vanneste S., et al, 2010 | 240 participants | Verification of the effect of transcutaneous electrical nerve stimulation (TENS) for somatosensory tinnitus treatment | Real and sham TENS treatment was applied for 30 minutes. VAS for tinnitus loudness used as outcome measure | Statistically significant reduction of tinnitus (p<0,001); 43/240 responded to TENS with a 42,92% improvement |
| LatifpourD.H., et al, 2009 | 24/41 participants (12 men, 12 women; age: 18-70 years) | efficacy of strecthing, postural training and acupunture in patients with somatosensory tinnitus | 2 groups (intervention and waiting list); outcome measures: VAS, HADS, Klockhoff test, mobility of neck and posture; assessment at pre and post treatment and at 3 months follow-up | statistically significant improvement in VAS pre-post treatment (p<0.001) and at follow up (p<01) and at Klockhoff test pre-post treatment (p<0,001) and at follow up (p<0.01); physical therapy components indicate a useful treatment of somatosensory tinnitus |
| Michiels S., 2016 | 38 participants (2 groups immediate-start and delayed start therapy group (n=19) | Investigate the effect of a multimodal cervical physical therapy treatment on tinnitus complaints in patients with somatosensory tinnitus | 2 groups immediate-start(n=19) and delayed start therapy group (n=19) underwent a 6 weeks physiotherapy treatment; outcome measures: Tinnitus Functional Index (TFI), Neck Bournemouth Questionnaire (NBQ) measures at baseline after wait-and-see period at the delayed group, after treatment and after 6 months follow-up, Global Perceived Effect measured at all moments except baseline | All patients decrease significantly TFI (p<0,04) and NBQ (p<0,001) scores after treatment; NBQ scores remain lower after 6 months follow up (p<0,001); 53% of patients experienced an immediate improvement of tinnitus after treatment, an effect lasting for at least 6 weeks in 24% of them. Cervical Physical Therapy has a positive effect on subjective tinnitus complaints |
